# Genomic analysis of Anderson typing phages of *Salmonella* Typhimrium: towards understanding the basis of bacteria-phage interaction

**DOI:** 10.1038/s41598-023-37307-6

**Published:** 2023-06-28

**Authors:** Manal Mohammed, Sherwood R. Casjens, Andrew D. Millard, Christian Harrison, Lucy Gannon, Marie Anne Chattaway

**Affiliations:** 1 Genomics and Infectious Diseases Research Group, School of Life Sciences, University of Westminster, 115 New Cavendish Street, London, W1W 6UW UK; 2 Division of Microbiology and Immunology, Department of Pathology, University of Utah School of Medicine, University of Utah, Salt Lake City, UT 84112 USA; 3 School of Biological Sciences, University of Utah, Salt Lake City, UT 84112 USA; 4 Department of Genetics and Genome Biology, University of Leicester, University Road, Leicester, LE1 7RH UK; 5 Gastrointestinal Bacteria Reference Unit, UK Health Security Agency, London, UK

**Keywords:** Genetics, Microbiology

## Abstract

The Anderson phage typing scheme has been successfully used worldwide for epidemiological surveillance of *Salmonella enterica* serovar Typhimurium. Although the scheme is being replaced by whole genome sequence subtyping methods, it can provide a valuable model system for study of phage-host interaction. The phage typing scheme distinguishes more than 300 definitive types of *Salmonella* Typhimurium based on their patterns of lysis to a unique collection of 30 specific *Salmonella* phages. In this study, we sequenced the genomes of 28 Anderson typing phages of *Salmonella* Typhimurium to begin to characterize the genetic determinants that are responsible for the differences in these phage type profiles. Genomic analysis of typing phages reveals that Anderson phages can be classified into three different groups, the P22-like, ES18-like and SETP3-like clusters. Most Anderson phages are short tailed P22-like viruses (genus *Lederbergvirus*); but phages STMP8 and STMP18 are very closely related to the lambdoid long tailed phage ES18, and phages STMP12 and STMP13 are related to the long noncontractile tailed, virulent phage SETP3. Most of these typing phages have complex genome relationships, but interestingly, two phage pairs STMP5 and STMP16 as well as STMP12 and STMP13 differ by a single nucleotide. The former affects a P22-like protein involved in DNA passage through the periplasm during its injection, and the latter affects a gene whose function is unknown. Using the Anderson phage typing scheme would provide insights into phage biology and the development of phage therapy for the treatment of antibiotic resistant bacterial infections.

## Introduction

Foodborne salmonellosis is an important concern for public health. It is caused by the enteric pathogen *Salmonella enterica*, which includes more than 2600 serovars. Although non-typhoidal *Salmonella* (NTS) serovars such as Typhimurium, Enteritidis and Dublin are predominantly associated with a self-limiting gastrointestinal illness, they have adapted to cause invasive diseases^1,2^. Human infection by invasive non-typhoidal *Salmonella* (iNTS) can result in serious systemic illnesses, bacteremia and focal systemic infections^3,4^. There is no licensed human vaccine against iNTS serovars; moreover, management of iNTS illness is complicated by the emergence of multidrug resistant strains^5^. Human outbreaks of NTS have been reported in several countries around the world including high-income countries^6–8^**.** It is therefore crucial to use accurate, reliable, and highly discriminative subtyping methods for NTS epidemiological characterisation and outbreak investigation. The Anderson phage typing scheme has been used worldwide for epidemiological surveillance of *Salmonella enterica serovar* Typhimurium (hereafter called *Salmonella* Typhimurium)^9^. However, this scheme is dependent on original Anderson stocks which will not last forever, and it is being replaced by the whole genome sequencing (WGS) subtyping methods^10,11^**.** Although Anderson phage typing system has become almost obsolete for typing of *Salmonella enterica* serovar Typhimurium, we showed earlier that it might provide a valuable model system for study of phage-host interaction^12–14^.

The Anderson phage typing scheme uses a unique collection of 30 specific *Salmonella* Typhimurium bacteriophages^9^. The system distinguishes more than 300 definitive phage types (DT) of *Salmonella* Typhimurium based on their patterns of lysis to the typing phages. These phages represent a historically unique combination of phages. Analysis of these phages by DNA hybridization and restriction fragment profiles revealed that they are derived from a small number of phages, and a majority of them are related to the well-known *Salmonella enterica* phage P22 and therefore, belong to the P22 cluster within the lambdoid phages^15^. In this report, we sequenced the Anderson typing phages and analysed their genome sequences. A longer-term aim is to use Anderson typing phages to understand the complex dynamics of bacteria-phage interaction through characterising the genetic determinants that are responsible for their differing host ranges. We also note phage typing is much cheaper and less technologically demanding than WGS determination, so it can still be useful especially in developing countries^16^. The Anderson scheme for *Salmonella* Typhimurium phage typing is problematic since it is dependent upon aliquots of the original lysates prepared by Anderson, that will not last forever and as we show below, even perfect reproduction of their procedures for making these phage preparations may not result in phages with the same properties (due to random recombination with prophages in the propagation strains). Our studies could provide a starting place for devising a reproduceable method for preparing better understood Typhimurium typing phages.

## Methods

### Phage and host strains propagation and DNA extraction

In this study, we used *Salmonella* Typhimurium DT36 (8M677) for propagation of the typing phages 8, 10 and 28, *Salmonella* Typhimurium DT4 (M1461) for the propagation of the phages 20 and 32, and *Salmonella* Typhimurium DT1 (8M302) for the propagation of typing phages 1, 2, 3, 4, 5, 6, 7, 11, 12, 13, 14, 15, 16, 17, 18, 19, 21, 22, 23, 24, 25, 26, 27, 29 and 35. Table 1 illustrates the pattern of reaction to typing phage set in host strains. Luria–Bertani (LB) medium was used for bacterial growth and phage propagation. Phage DNA was extracted as previously described^17^.

**Table 1:**
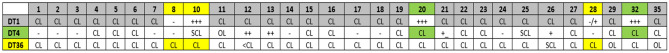
Pattern of reaction to typing phages in host strains DT1, DT4 and DT36.

Top row includes typing phages and left columns are host strains.

CL, confluent lysis; OL, opaque lysis; SCL, semi-confluent lysis; +++, 71–100 plaques; ++, 21–70 plaques; + , 5–20 plaques; −, no reaction; ± , a possible range of reactions between − and + .

The three bacterial host strains (DT1, DT4 and DT36 were cultured on nutrient agar media and incubated overnight at 37 °C. Genomic DNA was then extracted using QIAamp^®^ DNA Mini kit (Qiagen) according to manufacturer’s instructions. DNA quality and quantity were checked by gel electrophoresis and Qubit^®^ quantification platform (Invitrogen), respectively. Twenty 20–50 ng of DNA from each isolate was submitted for Illumina sequencing by Microbes NG.

### Genome sequencing of Anderson typing phages and bioinformatics sequencing analysis

Genomic DNA libraries of Anderson typing phages were prepared using the Nextera XT library preparation kit (Illumina, San Diego, CA, USA), following the manufacturer’s protocol. Genome sequencing of multiplexed libraries was carried out on the Illumina HiSeq platform using a 250-bp paired-end (PE) protocol^18^. Due to the coverage of sequencing data obtained, reads were first subsampled to lower coverage using a previously described method^19^. Briefly, phage genomes were assembled with SPAdes v3.12.0—only assembler option. As NexteraXT was used for the library preparation step, the termini of genomes could not be determined, therefore they were co-linearised against the reference *Salmonella* Typhimurium phage P22 (accession number: NC_002371.2) using *terS* as the first gene as recommended^20^. Genomes were checked for assembly errors using Pilon^21^. BAM files for input into Pilon were generated by the mapping of reads with bbmap.sh and processing files with SAMtools v1.10^22^, subsequent rounds of Pilon polishing were carried out until no further assembly errors were detected. Genome annotation was carried out using Prokka v1.14.6 using a custom HMM database based on the PHROGs^23^. Genomes were compared against a database of all known complete phages using the INPHARED database and associated scripts^24^.

Comparative analysis of phage genomes was carried out using VIRIDIC^25^. With comparative genomic analysis carried out with Roary^26^. Genomic open reading frame (ORF) map alignments were generated and visualised with Clinker^27^. Phylogenetic analysis based on the nucleotide sequence of *terL* was carried out with IQ-Tree using the following settings “-bb 1000 -m GTR”. The resultant tree was visualised and edited in iTOL^28^. Dotplot comparisons were created by Gepard^29^ and DNA Strider^30^.

### Genome sequencing of host strains and identification of plasmids, prophages, and restriction-modification and CRISPR-Cas systems

Genomic DNA libraries were prepared using a Nextera XT library preparation kit (Illumina, San Diego, USA) following the manufacturer’s protocol, and DNA of *Salmonella enterica* strains DT1, DT4 and DT36 was sequenced by MicrobesNG. WGS of multiplexed libraries was carried out on the Illumina HiSeq platform using a 250-bp PE protocol^18^. Bacterial genomes were assembled with SPAdes v3.12.0 and genome annotation was carried out using Prokka v1.14.6^23^. Identification of plasmids harboured by host strains was carried out using PLSD^31^ and the analysis was completed using Mash (search strategy: mash screen) with a maximal p-value of 0.1 and minimal identity of 0.99 and the winner-takes-all strategy was applied to remove redundancy from the output data. The identification and classification of the restriction–modification (R–M) systems were performed using Restriction–Modification Finder 1.1, REBASE^32^. A threshold of 95% was selected for minimum percent identity (%ID) between the sequence in the input genome and the restriction enzyme gene sequence; the selected minimum length was set at 60%. Prophages integrated into the genomes of host strains were determined using the web-based tool Phage Search Tool Enhanced Release, PHASTER^33^, applying default parameters. Detection of CRISPR-Cas systems was performed using CRISPRCasFinder^34^.

### Accession numbers

The sequence data is submitted to European Nucleotide Archive (ENA) under project number PRJEB48030.

### Informed consent

Statement was obtained from all subjects involved in the study.

## Results

### Properties of Anderson phages sequenced in this study

**Figure 1:**
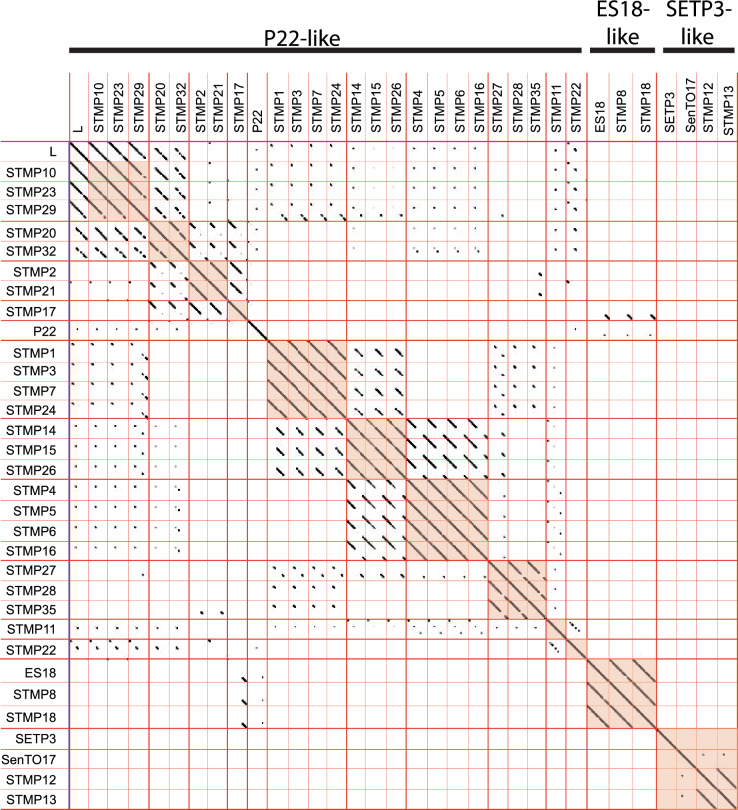
Whole genome dotplot analysis of Anderson typing phages. Dotplot of similarly oriented whole genome sequences were constructed with Gepard^29^ using a stringent word size setting of 1000; genomes are oriented with terminase at the left and lysis genes at the right end (see legend to Fig. 4 below). Thick red lines separate subclusters and clusters, and thin lines separate phage genomes. Groups of more highly related phages are indicated by an orange background. Phage names are shown on the left and top of the plot; phage cluster names are indicated above. Previously characterized phages P22, L, ES18, SETP3 and SenTO17 are shown for comparison (SETP3 has similarity to others in its group in lower stringency plots; Supplementary Figs. S2 and S3).

We sequenced the 28 of the 30 phages in the Anderson *S. enterica serovar* Typhimurium typing scheme (despite several attempts, we could not assemble typing phage 19 and 25 sequences as their raw reads were of poor quality). These phages have gone by various names, and in this report we name these phages “STMP” (for *Salmonella* Typhimurium typing phage) with their typing phage number. The genome sequences of these phages range from 39,289 to 47,070 bases in length. Initial analysis of the genomic similarities among the 28 phages by average nucleotide identity (ANI) and by genome dotplot agreed well and supported the notion that there are many similarities among the STMP phages. Figure 1 shows a genome dotplot, and Supplementary Fig. S1 shows a heatmap of the ANI values from VIRIDIC analysis^25^. A comparison of the Anderson typing phage genomes against all extant phage genomes by MASH^24^ showed that their best matches were to phages in three previously characterized groups, the P22-like, ES18-like and SETP3-like phage clusters defined by Grose and Casjens^35^ (Supplementary Table S1; summarized in the rightmost column of Table 2).

**Table 2:** Types of Anderson phages.

Phage types (subclusters)	Cluster	Repressor type^a^	Most similar phages^b^
STMP1, 3, 7 & 24 (3 & 24 identical)	P22-like	ST104	ABTLsp11242; S9-5
STMP2 & 21	P22-like	ST104	VSe13 (2); ST104 (21)
STMP4, 5, 6 & 16	P22-like	MG40	L, ST64T
STMP10, 23 & 29	P22-like	L	L, ST64T
STMP11	P22-like	ST104	SE1
STMP14, 15 & 26 (all three identical)	P22-like	ST104	SE1
STMP20 & 32	P22-like	L	L, ST64T
STMP22	P22-like	ST104	SE1
STMP27, 28 & 35	P22-like	P22 (28); ST104 (27 & 35)	SPN9CC
STMP17	P22-like^c^	P22	P22, VSe13
STMP8 & 18	ES18-like	P22/ES18^d^	ES18
STMP12 & 13	SETP3-like	None	TS6; SenTO17

^a^Deduced from their very high sequence similarity—ST104^66^, MG40^67^, P22^68^**,** L^69^. They all have point mutant differences from the repressors of these previously characterized phages, so it is not possible (except in cases of frameshift mutations and truncations) to determine if the typing phage repressors are functional.

^b^As calculated by MASH (Supplementary Table S1).

^c^The right end of STMP17 is very ES18-like. This phage is apparently a hybrid between an ES18-like phage and a P22-like phage.

^d^Phages ES18 and P22 have the same repressor specificity^39^.

### Taxonomy of Anderson typing phages

To compare the genomes of the typing phages in more detail, Gepard^29^ was used to generate low resolution genome dotplots that have high, medium and low scan window stringency (Gepard word settings 1000, 100 and 10) are shown in Fig. 1 and Supplementary Figs. S2 and S3, respectively. In these plots and others presented in this report, all the genomes are oriented so that the terminase genes are at the left end and lysis genes are at the right end (the standard lambdoid phage orientation). The low and medium stringency dot plots show that the 28 phages clearly fall into the three major types indicated by MASH (as mentioned above) (highlighted in yellow in Supplementary Figs. S2 and S3). These three phage types correspond to three previously known phage clusters or genera (Table 2). (i) A majority of the Anderson typing phages are P22-like [called the “P22-like cluster” by Grose and Casjens^35^ and the genus *Lederbergvirus* by the International Committee on Taxonomy of Viruses (ICTV)]. In addition, two of the typing phages, (ii) STMP8 and STMP18 are similar to the previously described phage ES18^36^ (the ES18-like cluster has not been assigned a genus name by ICTV), and (iii) two phages, STMP12 and STMP13, are similar to the SETP3-like phage cluster^37^ (also called ICTV genus *Cornellvirus*); we note that the weak similarity between the P22-like and ES18-like groups in the low and medium stringency dotplots is due to the fact that they are both lambdoid phages that have very different virion assembly genes but have some similarities among their early genes. The typing phage relationships with P22 and ES18 were deduced by Schmieger^15^ through DNA hybridization and analysis of restriction fragment patterns of phage virion DNA. P22-like and ES18-like phages are temperate^38,39^; however, the SETP3-like phages are virulent and unable to form lysogens^37^. BLASTn^40^ analysis of the current sequence database showed that typing phages STMP12 and STMP13 closest relatives are *Salmonella enterica* serovar Typhimurium phage SenTO17^41^; (accession number: MT012729) and TS6 (accession number: MK214385). These two phages are both members of subcluster E of the SETP3-cluster as defined by Casjens et al*.*^42^, but STMP12 and STMP13 are in fact sufficiently distantly related to be prototypes of a new subcluster. Interestingly, some of the first SETP3-like phages whose genome sequences were determined, including that of SETP3 itself, are members of the *Salmonella* Enteritidis typing phage set^37^. The high stringency dotplot of the Anderson typing phages shows that these three clusters naturally separate into twelve “subclusters” (highlighted in orange in Fig. 1) that have strong diagonal similarity lines indicating high sequence homology and largely syntenic genomes (summarized in Table 2).

### The P22-like typing phages

**Figure 2:**
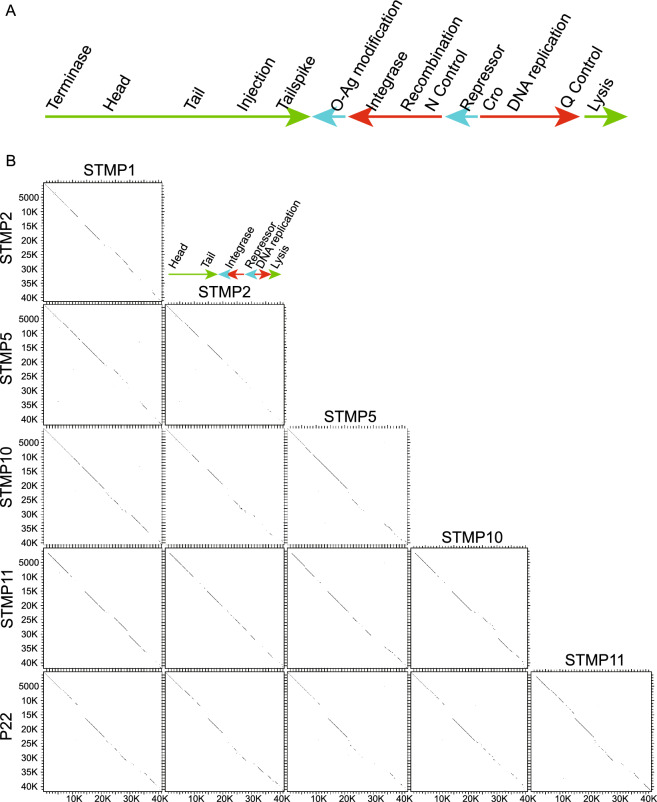
High resolution dotplots of selected P22-like Anderson typing phages. (**A**) Map of phage P22 genome. Arrows indicate the major transcripts; red, early genes; green, late genes; blue, genes expressed in a lysogen^38^. Functional module locations are shown above the transcripts. (**B**) Dotplots of similarly oriented typing phage genome sequences were constructed with DNA Strider^30^ using a stringency of 15 matches in a 15 bp scan window; all genomes are oriented as in part A of this figure (indicated by colored inset). Thick lines separate clusters, and thin lines separate phage genomes. Phage names are shown on the left and top of the plot, and scales are shown on the left and below the plots.

**Figure 3:**
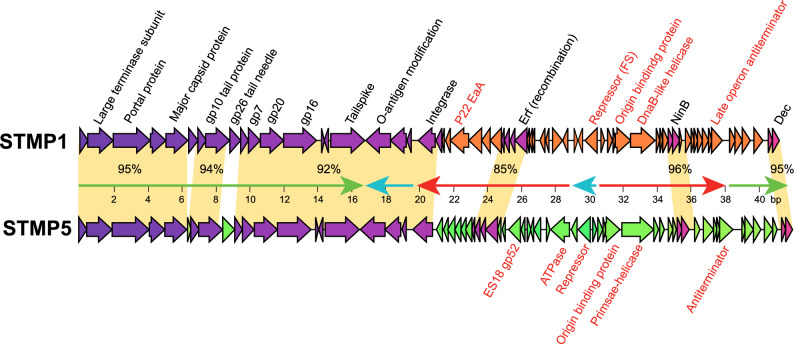
Genes in mosaically related STMP1 and STMP5 genomes. Genes are shown as thick arrows that indicate their direction of transcription. Genes of the same color are highly similar and orange regions between the maps indicate the major highly similar regions; nucleotide % identity values are shown for these sections. “gpX”, homologue of P22 gene X; FS, contains frameshift mutation. The major transcripts are shown as thin arrows; colors same as in Fig. 2.

The dotplots in Fig. 1 show at low resolution that the ten different P22-like Anderson phage subclusters are mosaically related to one another, and Supplementary Fig. S4 shows this with comparative gene maps. None of the typing phages corresponds perfectly to a previously known phage with a sequenced genome, and they have substantial differences from P22, the prototype for the P22-like phage cluster^35^. The genome mosaicism among the subclusters and between them and phage P22 is exemplified in the higher resolution dotplots comparing five subclusters, those typified by STMP1, STMP2, STMP5, STMP10 and STMP11, in Fig. 2; the other five subclusters have similar but different relationships. Figure 3 shows a typical the mosaic relationship, that between STMP1 and STMP5, in terms of the genes present. Most subclusters with more than one member also have some intra-subcluster genome mosaicism with smaller indel relationships; only subclusters typified by STMP4 and STMP14 have no intra-subcluster mosaicism. These subclusters are in general consistent with and greatly extend Schmieger’s^15^ identification of EcoRI fragment pattern types; however, our STMP15 and STMP16 sequences do not agree with his placement of these phages in the same group as STMP5 and STPM10, respectively.

The specific relationships among the ten P22-like subclusters are complex and not described in detail here, but we mention a few of their differences as examples. The virion assembly proteins of these phages, which are encoded by the left half of the genome (as displayed in “standard orientation” in Fig. 2A) are generally very similar to those of P22 indicating that their virions are short tailed and very similar to that of P22. The differences among the P22-like typing phage genomes are largely in the early region (right half) genes. The small number major differences in the left half are as follows: (i) the small and large terminase of STMP11 are quite different from those of the other phages, with the encoded proteins being only about 15% and 45% identical, respectively, to those of the other P22-like typing phages. This terminase sequence type has not been observed in previously characterized phages, but nearly identical large terminase subunits are present in P22-like prophages in sequenced *Salmonella enterica* genomes (for example, 99.8% identical in serovar Typhimurium strain 877,363, accession number: AAPPZE010000017). (ii) The injection proteins (detailed function is mentioned below) encoded the subclusters typified by STMP2 (and STMP17 and STMP20) and by STMP11 (and STMP22) from two types that are quite different from the others. For example, the STMP2 and STMP11 gene *16* proteins (gp16s) are 35% and 28% identical to those of STMP1, respectively, and 50% identical to each other. The injection proteins are among the most variable virion assembly proteins^43,44^, and the best matches to previously characterized phages for the three typing phage gp16 types are as follows: STMP1 gp16 is 99% identical to phage SPC9CC^45^; (accession number: NC_017985), STMP2 gp16 is 92% identical to P22-like phage UPF_BP1 (accession number: KX776161) and the STMP11 gp16 is 100% identical to P22-like phage ST160^46^ (accession number: NC_014900).

We note that the receptor-binding proteins (tailspikes) of the Anderson P22-like typing phage virions are all ≥ 99% identical to the P22 gene *9* tailspike protein. P22 tailspikes bind the *Salmonella enterica* serovar Typhimurium host’s O:4 type surface O-antigen polysaccharide^47^, so these typing phages certainly also use O:4 O-antigen as receptor. This similarity of the tailspikes of the P22-like (and SETP3-like, more details below). Anderson typing phages suggests that binding of the phage virions to their primary receptor is not a discriminating factor among these 26 phages in the typing scheme.

The P22-like typing phage early genes are considerably more variable than the late genes (Supplementary Figs. S2, S3 and S4). A few examples are as follows: (i) Their integrases are all > 95% identical to that of P22 suggesting the ability to integrate into the host *thrW* tRNA gene, except for the STMP2 and STMP17 subclusters, which are 100% identical to integrase of P22-like phage SI8 (accession number: MK972688) and only about 16% identical to P22 and the other P22-like STMP integrases. The integration site of SI8 is not known. (ii) The P22-like typing phages exhibit five very different prophage repressor types (and thus five different operator specificities) as listed in Table 2. (iii) These phages exhibit three different sequence types of DNA replication gene modules. The STMP1, 10, 11, 14 and 20 subclusters (and only STMP27 in its subcluster) are closely related to each other and carry a divergent P22 type DNA replication module that encodes an origin binding protein that is a distant relative of phage lambda gene *O* origin binding protein that is 15% identical to it P22 gene *18* homologue and a DnaB-like helicase that is 29% identical to that of P22 gene *12* protein. The STMP2 and 21 subclusters (and 28 and 35 of the STMP27 subcluster) encode a second type of distant O protein homologue that is only 19% and 35% identical to the homologues of STMP1 and P22, respectively, and a DnaB-like helicase that is 30% and 98% identical to those of STMP1 and P22, respectively. The STMP5 and 17 subclusters encode a third type of very distant relative of lambda gene O protein, and instead of a DnaB-like helicase they encode a nonhomologous primase-helicase protein. Both the latter proteins are very similar to the primase-helicases of phages ES18, STMP8 and STMP18.

### The ES18-like STMP phages

STMP8 and STMP18 are members of the ES18-like cluster (as mentioned above) and are in fact very close relatives of ES18, as is expected since Kuo and Stocker^39^ isolated ES18 as a single plaque of Anderson typing phage STMP18. STMP18 has 20 bp differences and one 27 bp insertion difference from the reported ES18 genome sequence^48^ (accession number: AY736146). This high similarity indicates STMP8 and STMP18 have virions with long non-contractile tails like ES18^48^ and have repressor specificity that is the same as ES18 (which is the same as that of phage P22^39,48^. ES18 utilizes the FhuA outer membrane protein as receptor^49^, and its gene *29* central fiber protein (a homologue of phage lambda gene *J* protein) which presumably binds this receptor is identical to those of typing phages STMP8 and STMP18, so the latter certainly use this same receptor, and receptor binding by these phages separates them from the other phages in the Anderson typing scheme.

### The SETP3-like STMP phages

Comparative genomic analyses of STMP12 and STMP13 revealed that they are members of the SETP3-like phage cluster where they form a novel subcluster (as mentioned above). These phages have long, non-contractile tails and are not temperate, but have not been studied in detail. The SETP3-like phage SenTO17 (above) encodes a tailspike whose receptor binding domain is 98% identical to those of STMP12 and STMP13, and these are all about 62% identical to the P22 tailspike; this is the only homology between these two phage clusters. This level of similarity strongly suggests that STMP12 and STMP13, like the P22-like typing phages, also utilize the O:4 O-antigen as their cellular receptor^50^, and that host range differences between STMP12 and STMP13 are not due to receptor binding.

### Origins and lineages of the Anderson typing phages

**Figure 4:**
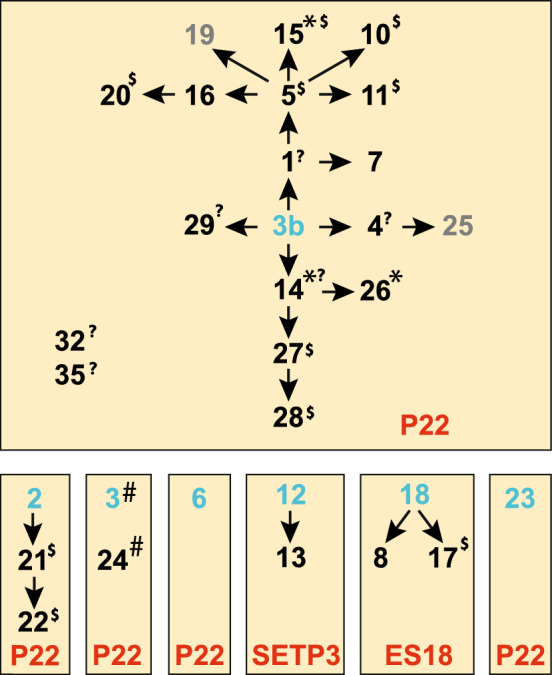
Anderson *Salmonella enterica* serovar Typhimurium typing phage origins. Blue and black numbers indicate STMP phages (blue are independent starting points for isolation of members the phage set; those in gray do not have available genome sequences), and orange boxes enclose phages that were independently isolated and arrows indicate the isolation of “modified” phages. The phage type for each box is indicated in red text. Asterisks (*), hashtags (#) and daggers (†) denote three sets of identical genomes. Dollar signs ($) mark genomes that have suffered significant (≥ 1 kbp) insertions, deletions or replacements relative to their immediate precursors, and question marks (?) indicate that it is not known if the phage has an indel relative to its immediate precursor since precursor sequence is not known. This figure was inspired by Fig. 1 in Schmieger^15^ and Fig. 1 in Rabsch^53^.

The origin of the Anderson typing phages is complex and somewhat murky^15,51–53^**.** The typing phage set includes at least seven apparently independently isolated phages and their “modified” derivatives. These modifications were the result of forced passages through various *Salmonella enterica* serovar Typhimurium isolates and subsequent screening for new host range properties^15^. A complex series of modifications of *Salmonella* Paratyphi B phage 3b resulted in eighteen of the typing phages, and six other independent phage isolates, STMP2, STMP3, STMP6, STMP12, STMP18 and STMP23, gave rise to six additional modified phages (we are unsure of the history of STMP32 and STMP35). The history of the sequenced Anderson typing phages was deduced by Schmieger^15^ and Rabsch^53^ and is summarised in Fig. 4. Schmieger^15^ and Rabsch^53^ suggested from restriction fragment analysis of phage virion DNAs that some of these modifications may be the result of recombination with resident prophages and of DNA methylation by R–M systems in the host strains used to propagate them. Our genome sequences support both of these notions in different cases.

The typing phage genome sequences are largely, but not simply and perfectly explained by their putative historical lineages (Fig. 4). Not all of the typing phage genomes fit the putative lineages. Of the 21 phages we can analyse in this context, there are five cases of very similar phages that are not the result of contiguous steps in the modification process. (i) STMP15 is identical to STMP14 and STMP26 but it is not contiguous to them in the Fig. 4 lineage. (ii) STMP6 is very similar to STMP4, STMP5 and STMP16 but is supposedly the result of an independent isolation, and (iii) in the same subcluster STMP4 modification step is not contiguous with the others. (iv) STMP10 and STMP23 are very similar but not derived from contiguous modification steps as STMP23 is the result of an independent isolation. (v) STMP1 and STMP7 are very similar but the other two subcluster members STMP3 and STMP24 are the result of an independent isolation. It is not known if these apparent incongruities are the result of incorrect reconstruction of the historical lineages, takeover by resident prophages (as discussed below), recombination with similar prophages in different propagation strains, or cross-contamination during the modification steps.

**Figure 5:**
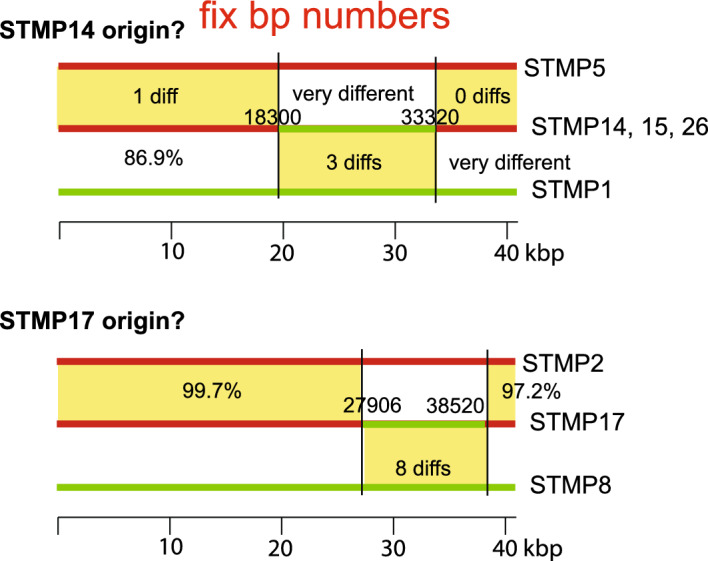
Examples of P22-like typing phage relationships. Colored horizontal lines represent genomes of closely related typing phages and shown with genomes oriented with terminase at the left and lysis genes at the right end as shown in Fig. 2. The extent of segment differences are shown in the yellow boxes and bp segment boundaries are indicated for the middle phages.

Of the ten modification steps from progenitor phage 3b where parent and derived phage genomes can both be analysed, seven derived phage genomes have suffered apparent DNA replacements relative their immediate precursor phage (i.e., parent and modified phage have mosaically related genomes). Similarly, two of the three modifications in the STMP2 lineage in Fig. 4 resulted in mosaic differences, as did one of the two steps in the STMP18 lineage. These findings, that ten of sixteen modification steps gave rise to phages with genetic replacements, strongly suggest that recombination with resident prophages or (less likely) contaminating free phages occurred during these modifications. As expected from these findings, there are numerous relationships among the ten subcluster genomes. For example, partial genome similarities in Fig. 1 indicate that the subcluster STMP15 phages appear to be possible hybrids of subcluster STMP1-like and STMP4-like phages, and STMP17 appears to be a hybrid of subcluster STMP8-like and STMP2-like phages. This is shown diagrammatically in Fig. 5. It is unclear exactly how these relationships originated.

In other P22-like typing phage modification steps, STMP26 derivation from STMP14, and STMP24 derivation from STMP3, there was no nucleotide sequence change. It is likely that the DNA of the members of each of these pairs was modified by host methylation differently so that restriction systems in *Salmonella* isolates being phage typed deal with them differently. The fact that STMP15 is identical to STMP14 and STMP26 is curious since it is supposedly separated by several independent modifications in the Fig. 4 scheme (as mentioned above). We have no simple explanation for this observation.

**Figure 6:**
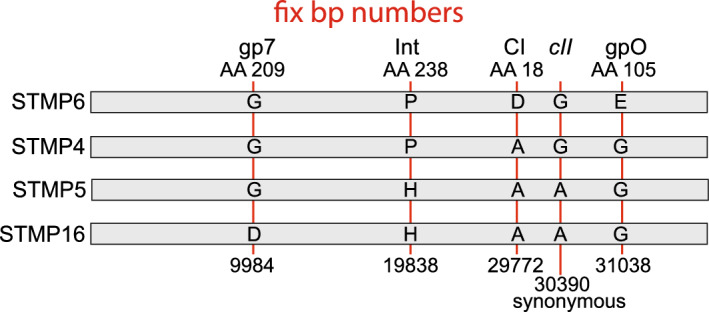
Single bp differences among the STMP4 subcluster phages. Amino acids (or nucleotide in the case of the synonymous different in the c*II* gene) are shown for the indicated positions are shown on genome maps (approximately to scale). Nucleotide numbers are shown below.

Other typing phage modifications resulted in phages with no large indels relative to one another. The P22-like typing phage with only a few bp differences are as follows: (i) STMP1 and STMP7 differ by only 5 bp—2 bp and 1 bp differences in the major capsid protein gene that change amino acids 43 and 402 in the encoded protein, and a 1 bp deletion and a 1 bp substitution in the repressor gene that cause a translational frameshift and change amino acid 37 (translationally upstream of the above frameshift), respectively. It is curious that the apparently intact repressor in STMP7 is the result of modification of STMP1 whose repressor gene is inactive, but agrees with the fact that Schmieger^15^ reported that “A1” (STMP1) makes clear plaques and “A7” (STMP7) makes turbid plaques. It is unclear how repressor gene inactivation could affect host range, since phages with intact or mutant repressor should both be immune to a host with a repressor of the same specificity, so it is unclear how these differences can affect host range. (ii) Phages STMP4, STMP 5, STMP6 and STMP16 are very similar but have different combinations of four single bp differences that cause amino acid differences in the injection protein homolog of P22 gene 7 protein (gp7), CI repressor (here and elsewhere in this report we use the phage lambda, rather than the P22 nomenclature for the genes that control lysogeny), integrase and gpO origin binding replication protein as well as a synonymous codon change in the *cII* transcription activator gene as is illustrated in Fig. 6. The above changes in CI and integrase may change ability to lysogenize and thus alter plaque clarity, but it is not obvious how they would affect host range, and it is not possible to predict the effects of the gpO change or the synonymous change in *cII*. (iii) Only one pair among these four phages, STMP5 and STMP16, have genomes that differ by only a single bp, and this difference lies in the gene that is the homologue of P22 gp7 which is involved in DNA injection. During injection P22 DNA travels from the virion to the cytoplasm of the infected cell through a conduit that is built from gp7, gp16 and gp20 proteins that are released from the virion after adsorption, and gp7 is thought to form the virion proximal portion of this trans-periplasm conduit^54^. Since gp7 is presumably exposed to the periplasm, it may interact with host components there that could variably affect injection and thus host range. Nonetheless, since the DNA methylation status of the original typing phage stocks is not known, any host range differences could be mediated by methylation and restriction. Additional experimentation will be required to separate these possibilities.

The two ES18-like typing phages are also very similar. STMP8 resulted from a modification of STMP18 (Fig. 4), and they have five single bp differences as follows: (i) C to A at 4870 in ES18 gene *7* protein, the prohead protease that cleaves major capsid protein during head assembly; (ii) insertion of G at 9841 in ES18 gene *16* tail tube protein. This frameshift truncates the tail tube protein, but deletions of the C-terminual region do not always inactivate these proteins^55^; (iii) A to C at 33206 in ES18 gp48 single strand DNA binding protein that presumably acts in DNA replication and/or recombination; (iv and v) A to C at 34306 and C to T at 34368 in ES18 gp52 (homologue of lambda *cIII*) which helps establish lysogeny. It is not clear how these changes could affect host range.

The two SETP3-like typing phages, STMP12 and STMP13, have only one bp difference, a 1 bp insertion at bp 39590 in the modified phage STMP13 that causes a translational frameshift in the homologue of gene *52* of SETP3. The function of this gene, which lies between the replication and lysis genes, is not known, so no speculation on how it might affect host range can be made.

### Host genetic determinants contribute to host susceptibility to Anderson typing phages

In order to begin to understand the host differences that are responsible for *Salmonella* phage types, the sequences of the genomes of the different types will be required in addition to the typing phage sequences. We began this process by sequencing the genomes of *Salmonella* strains DT1, DT4 and DT36 on which the typing strains were propagated for this study (Table 1).

#### Prophages in propagation host strains

Analysis of their genomes by PHASTER^33^ indicated that the three host strains used for propagation of the Anderson typing phages, DT1, DT4 and DT36, carry several prophages that are listed in Table 3. All three have a Gifsy-2-like prophage phage and a prophage remnant that is similar to *Escherichia* phage 500465-1 (accession number: NC_049342). Gifsy-2 is a lambdoid phage with non-contractile long tails^56^ (accession number: NC_010393.1) and 500465-1 is P2-like phage with very little relationship to the lambdoid phages. It is very unlikely that the lambdoid P22-like or ES18-like typing phages could form viable recombinants with them. DT1 and DT36 harbour a prophage that is similar to phage 118970_sal3^57^ (accession number: KU92749) that is absent from DT4. Phage 118970_sal3 is a lambdoid phage with a long contractile tail whose virion assembly genes are most similar to *Salmonella* phage ST64B^58^ (accession number: AY055382). DT4 also carries a Fels-2-like prophage which is absent from both DT1 and DT36. Like 500465-1, Fels-2^59^ (accession number: NC_010463) is a P2-like phage and very unlikely to recombine with any of the typing phages. We screened the phages that were propagated on DT1 (STMP20 and STMP32) and DT36 (STMP8, STMP10 and STMP29) for homology to their host’s prophages, but found no close sequence similarities. We conclude that these phages did not recombine with the prophages in these strains and since there are no P22-like or ES18-like prophages in these host strains, it is very unlikely that recombination with resident prophages might have occurred during their propagation for our studies, so we believe that all the phages in this report faithfully represent the genome sequences of the original Anderson typing phages.

**Table 3:** Prophages in typing phage propagation host strains.

Prophage	Phage accession number	DT1	DT4	DT36
Gifsy-2-like	NC_010393	Intact (> 99% identity)	Intact (> 99% identity)	Intact (> 99% identity)
118970_sal3-like	NC_031940	Intact (> 99% identity)	Absent	Intact (> 99% identity)
Fels-2-like	NC_010463	Absent	Intact (100% identity)	Absent
500465-1-like	NC_049342	Degraded (22% coverage, > 83% identity)	Degraded (68% coverage, > 88% identity)	Degraded (22% coverage, > 83% identity)

% identity compared to reference phages.

#### Plasmids in host strains

We detected three identical plasmids among the genome of all three host strains (including P02 of *Salmonella* Typhimurium, pSE81-1705-3 of *Salmonella* Enteritidis and plasmid 3 of *Salmonella* Senfternberg) as indicated in Table 4. Interestingly, both DT4 and DT36 harbour two extra plasmids pSLT7_1 and pCFSAN008081 of *Salmonella* Typhimurium; however, those two plasmids are absent from DT1.

**Table 4:** Distribution of plasmids identified among the host strains genomes.

Plasmid	Accession number	Length (bp)	DT1	DT4	DT36
P02 of *S. Typhimurium*	NZ_OU015330.1	69,651	Present (> 99% identity)	Present (> 99% identity)	Present (> 99% identity)
pSE81-1705-3 of *S. Enteritidis*	NZ_CP018654.1	33,784	Present (> 99% identity)	Present (> 99% identity)	Present (> 99% identity)
Plasmid 3 *S.* of *S. Senftenberg*	NZ_LN868945.1	147,787	Present (> 99% identity)	Present (> 99% identity)	Present (> 99% identity)
pSLT7_1* S. Typhimurium*	NZ_CP064264.1	93,946	Absent	Present (> 99% identity)	Present (> 99% identity)
pCFSAN008081* S. Typhiumurium*	CP074664.1	93,960	Absent	Present (> 99% identity)	Present (> 99% identity)

% identity compared to the reference plasmid.

#### Restriction-modification systems in host strains

We detected four types of R–M systems (I, II, III, and IV) in the genomes of host strains as illustrated in Table 5. Interestingly, type I methytransferase *M.SenTFII* was found only in DT1 and DT4 but absent in DT36. On the other hand, DT36 harbours type I methyltransferase *M.Sen189911* which is absent from both DT1 and DT4. Interestingly, both DT1 and DT36 genomes harbour type II restriction enzyme/ methyltransferase; *StyUK1IV* which is absent from DT4 genome.

**Table 5:** Distribution of R–M systems in the host strains.

Gene	Type	Function	Recognition sequence	DT1	DT4	DT36
*M.SenTFII*	I	Methyltransferase	GAGNNNNNNRTAYG	Present	Present	Absent
*S.StyUK1II*	I	Specificity subunit		Present	Present	Present
*M.Sen189911*	I	Methyltransferase		Absent	Absent	Present
*M.StyUK1V*	II	Methyltransferase		Present	Present	Present
*M.SenAboDcm*	II	Methyltransferase	CCWGG	Present	Present	Present
*StyUK1IV*	II	Restriction enzyme/methyltransferase		Present	Absent	Present
*M.Sen641III*	II	Methyltransferase	ATGCAT	Present	Present	Present
*SenAZII*	III	Restriction enzyme	CAGAG	Present	Present	Present
*M.StyUK1I*	III	Methyltransferase		Present	Present	Present
*STyLT2Mrr*	IV	Methyl-directed restriction enzyme		Present	Present	Present

#### CRISPR-Cas systems in host strains

Two CRISPR loci, CRISPR-1 and CRISPR-2, were detected within the three host strains. Although host strains contain highly similar palindromic repeats each strain has its own spacers that vary in number and/or pattern as illustrated in Table 6.

**Table 6:** Distribution of CRISPR/Cas systems in host strains.

Strain	Cas cluster subtype	Spacer number		
		CRISPR-1 locus	CRISPR-2 locus	Total
DT1	I-E	40	11	51
DT4	I-E	23	28	51
DT36	I-E	22	15	37

Interestingly, spacer CCACGTTCGGCGATGTTGGCCCCATCGGTCCA present in DT1, DT4 and DT36 is found in typing phages 1, 7, 10, 14, 15, 20 and 26 but absent from other phages. While spacer TCTGGTTATAACATCGCAGCAAAATCAAAAGA detected in DT1, DT4 and DT36 is found only in phages 12 and 13. Spacer CCAGAAAGTGCCGGTAGTGCCTGATGAACGAC detected in DT4 and DT36 found in typing phages 10, 20 and 22. Further work will be required to know if CRISPRs are actually important in Typhimurium phage typing.

## Discussion

Much remains to be understood about the molecular basis of host-phage interactions. Using the Anderson phage typing scheme to understand the dynamics of host-phage interaction should provide insights into phage biology and the development of phage therapy as an alternative to antibiotics for the treatment of antibiotic resistant bacterial infections.

Although Anderson phages are closely related to each other they differ in their effect on the three host strains; *Salmonella* Typhimurium DT1, DT4 and DT36 (Table 1). For example, STMP phages 1, 2, 3, 7, 14, 17, 18 and 24 induce confluent lysis (CL) in host strains DT1 and DT36; however, DT4 is resistant to these phages. On the other hand, host strains DT1 and DT4 are resistant to STMP8, while DT36 is susceptible to STMP8. Interestingly, the 3 host strains are all susceptible to phages 4, 5, 6, 11, 15, 16, 19, 22 and 23. In this study, we applied WGS technology to investigate the genomic correlates of the striking difference in phage susceptibility among host strains; DT1, DT4 and DT36.

The genome sequences of the Anderson typing phages showed that they comprise three quite different types, P22-like, ES18-like and STEP3-like phages. These phages were derived from a small number of progenitors by forced passage through various *Salmonella* strains and subsequent isolation of phages with altered host range properties. Such “modifications” could in theory be of several types as follows: (i) changes in DNA methylation with no nucleotide sequence change, (ii) one or a few nucleotide changes due to mutation of the input phage or recombination with very similar parts of resident prophages, (iii) recombination with similar but mosaically resident prophages, and (iv) replacement of the input phage by an induced resident prophage in propagating host strain. The genome sequences show clearly that all of these may have occurred as follows: (i) Several sets of identical phages (STMP14/15/26 and STMP3/24) are present. (ii) Several sets of phages that differ by only a few point mutations (e.g., STMP4/5/6/16 and STMP12/13) are present. (iii) We found numerous examples of derivative phages that have sizable replacements relative to their mosaically related immediate precursor. These are most easily explained by recombination with resident prophages, and we note that Schmieger^15^ found that at least 19 of 25 Anderson phage propagation hosts tested harbour prophages that are related to the typing phages. We note that such hybrid phages form quite efficiently through homologous recombination between very similar sequences in mosaically related genomes^60^. (iv) It is quite possible that some of the cases of particularly different “modified” phages are the result of takeover by an induced prophage. For example, STMP11 has terminase genes and several early gene types that are unique among the typing phages (Supplementary Fig. S4), and Schmieger^15^ found that the STMP11 propagating strain has a prophage that is very similar to STMP11 by the low resolution techniques available at the time.

What differences are responsible for the unique host range properties of each of the Anderson typing phages? Perhaps surprisingly, with one possible exception these differences are not due to differences in adsorption to the host cell. There are only two different adsorption specificities among the 28 phages whose genomes were sequenced; 26 of the sequenced typing phages encode tailspikes that are so closely related to the P22 tailspike that it is essentially certain that they all adsorb with similar efficiency to the Typhimurium O:4 O-antigen surface polysaccharide, and two are identical to phage ES18 putative receptor binding protein (the central tail fiber) so they must utilize the host FhuA outer membrane protein as receptor. Our sequences show that most of the typing phages appear to have suffered recombination with resident prophages (or complete replacement by an induced prophage) during modification steps. For such phages the genetic differences are many so it is not possible to rigorously correlate their genomic differences with their plating properties. In only a few cases it is possible to rigorously deduce the genetic reason for the phages’ host range differences. In the two sets of phages with identical sequences (as mentioned above) it must be epigenetic differences that are responsible for their altered plating properties, and we suggest this is most likely DNA methylation by different classical R–M systems (and we note that R–M differences could also be responsible for host range variation among phages that are also very different genetically). Finally, in two cases phages differ by a single bp change that potentially identifies the gene responsible for the different plating properties. First, STMP12 and STMP13 differ by only a single bp deletion in the homologue of phage SETP3 gene *52*. This group of phages is not well-studied, and unfortunately nothing is known about the function of this gene, so the mechanistic reason for the host range difference remains unknown. Finally, phages STMP5 and STMP16 differ by a single bp in the homologue of P22 gene *7*. This protein is released from the virion after adsorption and then forms the virion proximal part of the conduit through which DNA passes on its way from the virion through the periplasm into the cytoplasm^54^. A difference in gp7 could alter its interaction with a host outer membrane or periplasmic protein, and if that interaction affects the assembly or function of the conduit it would affect DNA injection and thus host range. This is the first indication that gp7 may have such an interaction. The varied host range properties of the Anderson typing phages remain interesting since many of their differences appear to affect infection steps after adsorption that are incompletely understood; the gp7 effect above points out a previously unknown potential interaction that was discovered by our analysis in this report. However, dissection of their many genetic differences will require substantial further experimentation.

CRISPR-Cas systems are important bacterial antiphage defence systems, and they are considered to be the adaptive bacterial immune system that provides acquired immunity against invading phages^61^. These CRISPR repeat arrays contain interspersed “spacers” which provide information on the past exposure of the bacteria to foreign DNA including phages. Although host strains DT1, DT4 and DT36 contain highly similar palindromic repeats each strain has its own spacers that vary in number and/or pattern which could explain the difference in host cells’ susceptibility to Anderson phages. The high number of spacers in the CRISPR sequences, as well as their homology with bacteriophages suggest their possible role in bacterial resistance to invading phages. It was reported earlier that the conserved genetic organization of the *cas* genes in different *Salmonella* serovars including Typhimurium is consistent with the system having a biological function in these bacteria^62^. Interestingly, genomic analysis of Anderson phages revealed an absence of known anti-CRISPR (ACR) genes. The difference in bacterial susceptibility to phages could be linked to a phenomenon known as superinfection exclusion (SIE) where an existing prophage within bacterial genome prevents infection by other phages^63^. R-M systems also contribute to bacterial resistance to phages since they allow bacteria to recognize and destroy invading phages DNA by restriction endonucleases^64^. In this study, we detected four types of R-M systems (I, II, III, and IV) in the genomes of host strains and we found that several of the typing phages very likely differ in the methyl group modifications they carry, so R–M systems almost certainly play a part in phage typing. We detected different plasmids in the genomes of host strains. Acquisition of plasmids might be associated with the differences in phage susceptibility since a previous study^65^ showed the change between phage types in *Salmonella* Enteritidis was related to acquisition of a plasmid.

The interaction between phages and their bacterial hosts is complex. In addition to adsorption, injection and phage exclusion systems we discussed above, infecting phages interact with their hosts in numerous other important ways during infection. Since P22 and ES18 are members of the lambdoid temprate phage family whose various members encode proteins with similar functions, they have similar molecular lifestyles—including transcription patterns—to those of lambda. These phages do not encode an RNA polymerase or a DNA polymerase, so they are completely dependent upon these host enzymes for their gene expression and DNA replication. Phage lambda’s interaction with its host has been studied, and important phage protein–host interactions have been characterised that (i) recruit the host DNA synthesis machinery to the phage replication origin, (ii) control transcription initiation and termination, and (iii) allow host protein chaperones to participate in the folding of phage-encoded proteins. In addition, host DNA-binding proteins affect lambda DNA packaging during lytic growth and integration during establishment of lysogeny. These interactions have been reviewed earlier^70,71^. In addition, phage induced cell lysis depends on phage protein interactions with the host’s inner and outer membranes as well as the periplasmic peptidoglycan^72^. Figure 3 and Supplementary Fig. S4 show that the P22-like Anderson typing phages have considerable diversity in their genes that are involved in all of the above interactions. Thus, genetic variations among the Anderson host strains could differentially affect any of these critical processes as well as not-yet-discovered interactions. We believe that the Anderson typing phages represent a good model for studying the complex dynamics of phage-host interactions; and our current study represents an initial step toward understanding the molecular bases of such interactions. Further progress can certainly be attained by more detailed examination of the nature of the specific molecular defects in the various growth-restricted Anderson typing phage-host infections.

## Supplementary Information

Supplementary Information.

## Data Availability

The data presented in this study is available at ENA under project number PRJEB48030.
