# Supplementary material for: Genomic analysis of Anderson typing phages of Salmonella Typhimrium: towards understanding the basis of bacteria-phage interaction
Source: Sci Rep. 2023 Jun 28;13:10484. doi: 10.1038/s41598-023-37307-6 (PMC10307801; doi:10.1038/s41598-023-37307-6)
Supplement: Supplementary file 1 — Supplementary Information. [file 41598_2023_37307_MOESM1_ESM.pdf]

SUPPLEMENTARY Figures and Table

Figure legends

Figure S1. Heatmap of the whole genome similarities of the Anderson typing phages.
The average nucleotide identities (ANI) for all possible STMP phage genome comparisons are shown in the figure. Values range from 0 (0%) to 1 (100%); white represents 0% ANI and clusters of highly similar phages are highlighted in blue and green. The heatmap was produced using VIRIDIC.

Figure S2. Moderate stringency whole genome dotplot analysis of Anderson typing phages.
Dotplot of similarly oriented whole genome sequences were constructed with Gepard using a word size setting of 100; genomes are oriented with terminase at the left and lysis genes at the right end (see figure 2A of the text). Thick red lines separate subclusters and clusters, and thin lines separate phage genomes. Groups of more highly related phages are indicated by an orange background. Phage names are shown on the left and top of the plot; phage cluster names are indicated above. Previously characterized phages P22, L, ES18, SETP3 and SenTO17 are shown for comparison.

Figure S3. Low stringency whole genome dotplot analysis of Anderson typing phages.
Dotplot of similarly oriented whole genome sequences were constructed with Gepard using a word size setting of 11. See legend for figure S2 for figure details.

Figure S4. Gene maps of the Anderson typing phages.
The figure was created and genomes aligned by CLINKER with default settings; labels were added with Adobe ILLUSTRATOR. Predicted genes of the same color are very similar. The subclusters discussed in the text are marked with gray background boxes.

Figure S1

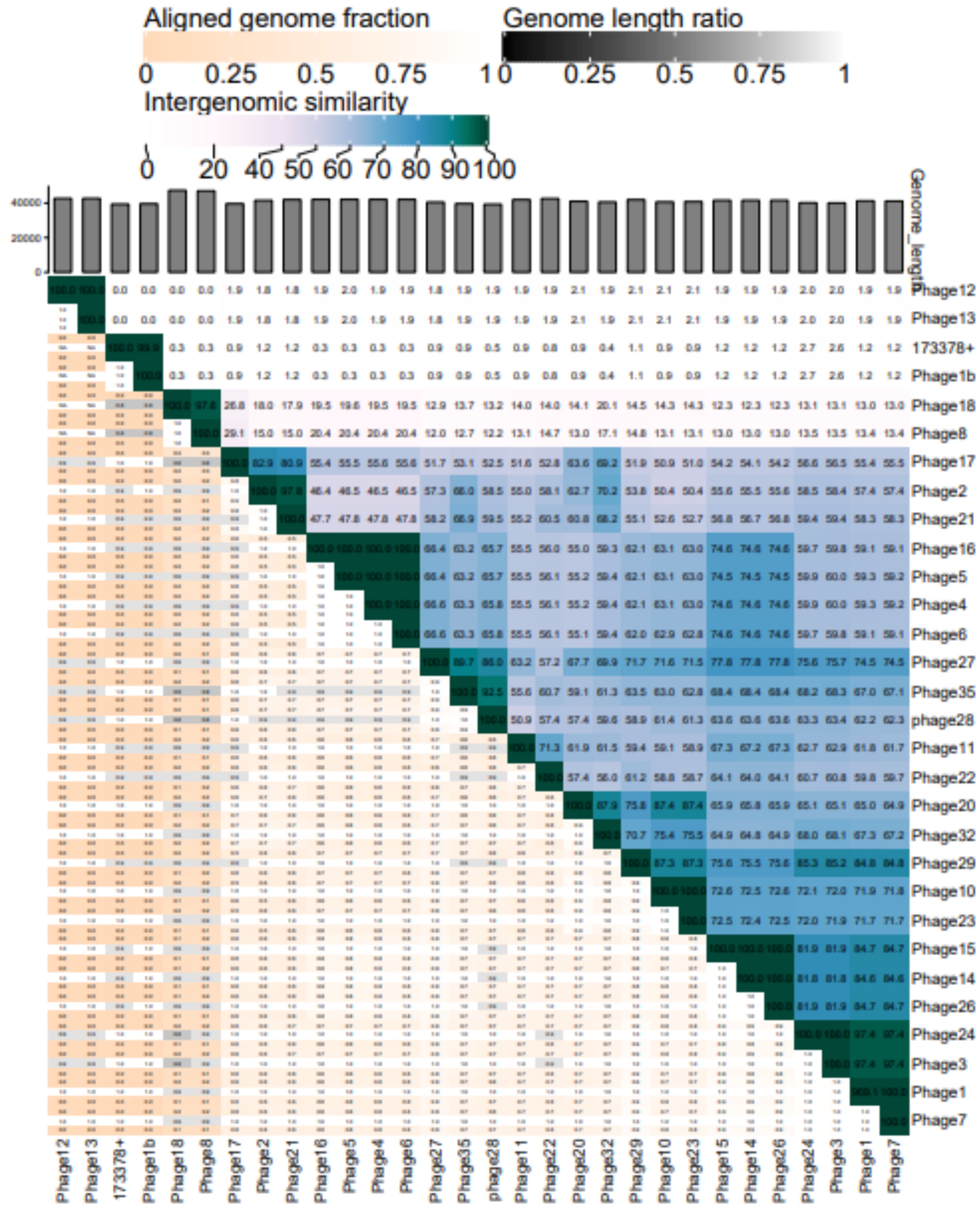

Aligned genome fraction
0
0.25
0.5
0.75
1
Genome length ratio
0
0.25
0.5
0.75
1
Intergenomic similarity
0
20
40
50
60
70
80
90
100
Genome_length
40000
20000
0
Phage12
Phage13
173378+
Phage1b
Phage18
Phage8
Phage17
Phage2
Phage21
Phage16
Phage5
Phage4
Phage6
Phage27
Phage35
phage28
Phage11
Phage22
Phage20
Phage32
Phage29
Phage10
Phage23
Phage15
Phage14
Phage26
Phage24
Phage3
Phage1
Phage7

Figure S2

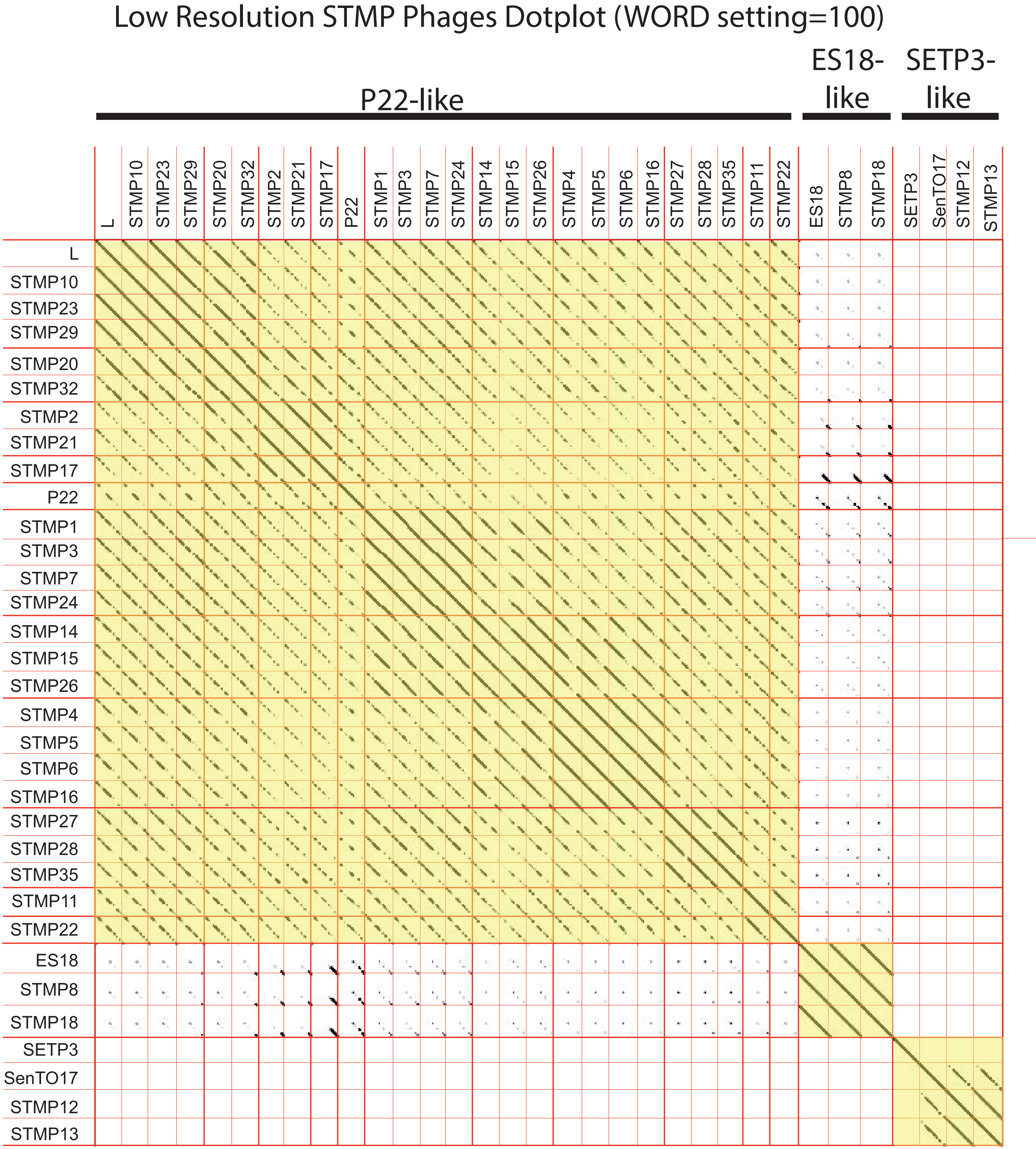
Low Resolution STMP Phages Dotplot (WORD setting=100)
P22-like
ES18-like
SETP3-like
L
STMP10
STMP23
STMP29
STMP20
STMP32
STMP2
STMP21
STMP17
P22
STMP1
STMP3
STMP7
STMP24
STMP14
STMP15
STMP26
STMP4
STMP5
STMP6
STMP16
STMP27
STMP28
STMP35
STMP11
STMP22
ES18
STMP8
STMP18
SETP3
SenTO17
STMP12
STMP13

Figure S3

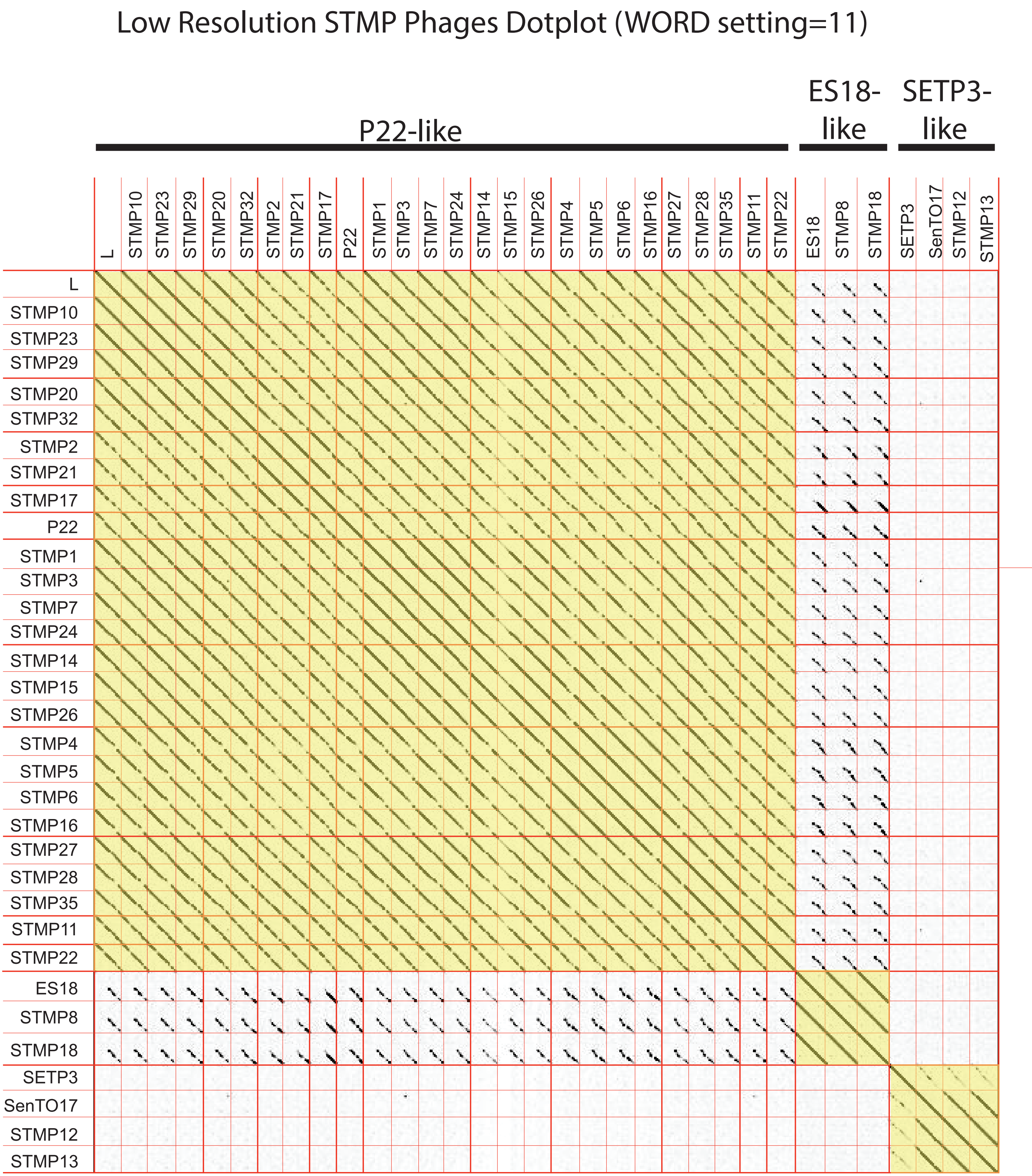
Low Resolution STMP Phages Dotplot (WORD setting=11)
P22-like
ES18-like
SETP3-like
L
STMP10
STMP23
STMP29
STMP20
STMP32
STMP2
STMP21
STMP17
P22
STMP1
STMP3
STMP7
STMP24
STMP14
STMP15
STMP26
STMP4
STMP5
STMP6
STMP16
STMP27
STMP28
STMP35
STMP11
STMP22
ES18
STMP8
STMP18
SETP3
SenTO17
STMP12
STMP13

Figure S4

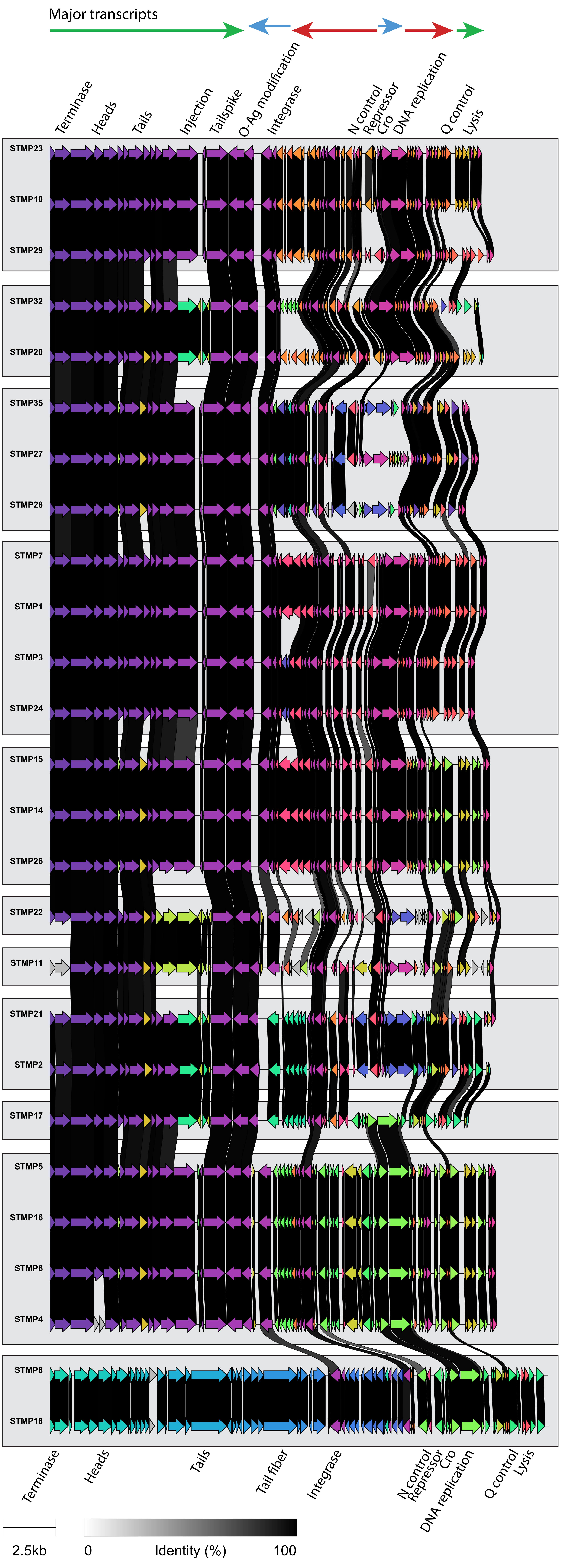

Major transcripts
Terminase
Heads
Tails
Injection
Tailspike
O-Ag modification
Integrase
N control
Repressor
Cro
DNA replication
Q control
Lysis
STMP23
STMP10
STMP29
STMP32
STMP20
STMP35
STMP27
STMP28
STMP7
STMP1
STMP3
STMP24
STMP15
STMP14
STMP26
STMP22
STMP11
STMP21
STMP2
STMP17
STMP5
STMP16
STMP6
STMP4
STMP8
STMP18
Terminase
Heads
Tails
Tail fiber
Integrase
N control
Repressor
Cro
DNA replication
Q control
Lysis
2.5kb
0
Identity (%)
100

Table S1. MASH closest phage matches to Anderson typing phages

Query	Hit (Acc.No)	Mash Distance	Identity(%)	Matching Kmers	Phage name	Genus	GC(%)	Length	Host
Phage1	MW790496	0.0254028	97.45972	415/1000	ABTNLsp11242	Unclassified*	48.177	40644	Salmonella
Phage1	MW722521	0.027077	97.2923	395/1000	S9-5	Lederbergvirus	48.163	39167	Salmonella
Phage2	MT932212	0.0515304	94.84696	204/1000	VSe13	Lederbergvirus	47.332	39265	Salmonella
Phage2	GQ422450	0.0519203	94.80797	202/1000	UAB_Phi20	Lederbergvirus	47.241	41809	Salmonella
Phage3	MW790496	0.0244475	97.55525	427/1000	ABTNLsp11242	Unclassified*	48.177	40644	Salmonella
Phage3	MW722521	0.0262266	97.37734	405/1000	S9-5	Lederbergvirus	48.163	39167	Salmonella
Phage4	MW013503	0.0378117	96.21883	292/1000	L cII-101	Lederbergvirus	47.514	40664	Salmonella
Phage5	MW013503	0.0378117	96.21883	292/1000	L cII-101	Lederbergvirus	47.514	40664	Salmonella
Phage6	MW013503	0.0376857	96.23143	293/1000	L cII-101	Lederbergvirus	47.514	40664	Salmonella
Phage6	MF188997	0.0378117	96.21883	292/1000	PM43	Lederbergvirus	47.461	40574	Salmonella
Phage7	MW790496	0.025484	97.4516	414/1000	ABTNLsp11242	Unclassified*	48.177	40644	Salmonella
Phage7	MW722521	0.0271635	97.28365	394/1000	S9-5	Lederbergvirus	48.163	39167	Salmonella
Phage8	AY736146	0.000191626	99.9808374	992/1000	ES18	Unclassified†	48.593	46900	Salmonella
Phage10	MW013503	4.76906e-05	99.99523094	998/1000	L cII-101	Lederbergvirus	47.514	40664	Salmonella
Phage10	AY052766	0.000119496	99.9880504	995/1000	ST64T	Lederbergvirus	47.523	40679	Salmonella
Phage11	DQ003260	0.0105261	98.94739	669/1000	SE1	Lederbergvirus	46.985	41941	Salmonella
Phage12	MK214385	0.0310744	96.89256	352/1000	TS6	Cornellvirus	51.131	41515	Salmonella
Phage12	MT012729	0.0368186	96.31814	300/1000	SenTO17	Cornellvirus	50.780	41658	Salmonella
Phage13	MK214385	0.0310744	96.89256	352/1000	TS6	Cornellvirus	51.131	41515	Salmonella
Phage13	MT012729	0.0368186	96.31814	300/1000	SenTO17	Cornellvirus	50.780	41658	Salmonella
Phage14	DQ003260	0.0301881	96.98119	361/1000	SE1	Lederbergvirus	46.985	41941	Salmonella
Phage15	DQ003260	0.0301881	96.98119	361/1000	SE1	Lederbergvirus	46.985	41941	Salmonella
Phage16	MW013503	0.0378117	96.21883	292/1000	L cII-101	Lederbergvirus	47.514	40664	Salmonella
Phage17	MT932212	0.0461689	95.38311	234/1000	VSe13	Lederbergvirus	47.332	39265	Salmonella
Phage17	AF217253	0.0482105	95.17895	222/1000	P22	Lederbergvirus	47.081	41724	Salmonella
Phage18	AY736146	0.00160079	99.839921	936/1000	ES18	Unclassified†	48.593	46900	Salmonella
Phage20	MW013503	0.0163866	98.36134	549/1000	L cII-101	Lederbergvirus	47.514	40664	Salmonella
Phage20	AY052766	0.0164427	98.35573	548/1000	ST64T	Lederbergvirus	47.523	40679	Salmonella
Phage21	AB102868	0.0414262	95.85738	265/1000	ST104	Lederbergvirus	47.431	41391	Salmonella
Phage21	MH370387	0.0414262	95.85738	265/1000	S149	Lederbergvirus	47.435	41391	Salmonella
Phage22	DQ003260	0.0171265	98.28735	536/1000	SE1	Lederbergvirus	46.985	41941	Salmonella
Phage23	MW013503	2.38274e-05	99.99761726	999/1000	L cII-101	Lederbergvirus	47.514	40664	Salmonella
Phage23	AY052766	9.55247e-05	99.99044753	996/1000	ST64T	Lederbergvirus	47.523	40679	Salmonella
Phage24	MW790496	0.0244475	97.55525	427/1000	ABTNLsp11242	Unclassified*	48.177	40644	Salmonella
Phage24	MW722521	0.0262266	97.37734	405/1000	S9-5	Lederbergvirus	48.163	39167	Salmonella
Phage26	DQ003260	0.0301881	96.98119	361/1000	SE1	Lederbergvirus	46.985	41941	Salmonella
Phage27	JF900176	0.00956826	99.043174	692/1000	SPN9CC	Lederbergvirus	47.326	40128	Salmonella
phage28	JF900176	0.00357808	99.642192	865/1000	SPN9CC	Lederbergvirus	47.326	40128	Salmonella
Phage29	MW013503	0.00676872	99.323128	766/1000	L cII-101	Lederbergvirus	47.514	40664	Salmonella
Phage32	MW013503	0.0272504	97.27496	393/1000	L cII-101	Lederbergvirus	47.514	40664	Salmonella
Phage32	AY052766	0.0274249	97.25751	391/1000	ST64T	Lederbergvirus	47.523	40679	Salmonella
Phage35	JF900176	0.00826969	99.173031	725/1000	SPN9CC	Lederbergvirus	47.326	40128	Salmonella

* P22-like (i.e., Lederbergvirus) by our bioinformatic analysis.
† ES18 typifies a cluster of phages not yet classified by ICTV.
